# Age-related changes of movement patterns in discrete Fitts’ task

**DOI:** 10.1186/1471-2202-14-145

**Published:** 2013-11-14

**Authors:** Rita Sleimen-Malkoun, Jean-Jacques Temprado, Eric Berton

**Affiliations:** 1 Aix-Marseille Université, CNRS, Institut des Sciences du Mouvement, UMR 7287 Marseille, France; 2 Aix-Marseille Université, Inserm, Institut de Neurosciences des Systèmes, UMR 1106 Marseille, France

**Keywords:** Aging, Discrete Fitts’ task, Behavioural dynamics, Movement patterns

## Abstract

**Background:**

Inspired by the framework of dynamical system theory, we aimed at exploring how the behavioural repertoire of the sensorimotor system can be reshaped with aging. Our reasoning was founded on the assumption that, with age, some of the existing patterns can be destabilized or even lost. In the present paper, this issue was investigated through the study of age-related changes in the movement patterns that are used to perform a discrete Fitts’ task. We analysed the performance of two groups of participants (young and older adults) across nine task difficulty levels, obtained via manipulation of target width.

**Results:**

Two movement patterns were revealed by the fact that increase in the index of the difficulty (ID) was accommodated through either the lengthening of both acceleration (AT) and deceleration (DT) times (*co-variation* pattern), or only DT (*dissociation* pattern). Analysis of the discontinuity in ID-AT relation showed that young participants switched from the *co-variation* to the *dissociation* pattern as ID increased. Pattern switching was accompanied by concomitant changes in the variability of AT/DT ratio. Older adults, on the other hand, used the *dissociation* pattern regardless of the ID. Consequently, they showed neither an abrupt discontinuity in ID-AT relation nor significant changes in the variability of AT/DT ratio across difficulty levels. Though the *dissociation* pattern was adaptive in young adults for high accuracy constraints, in older adults, it compromised task performance for lower difficulty levels.

**Conclusion:**

These findings support the view that aging may result in a compression of the neuro-behavioural repertoire. In sensorimotor tasks, it can lead to a loss of multi-stability in terms of available movement patterns, thereby compromising the ability of the neuro-musculo-skeletal system to adapt and face task constraints.

## Background

Understanding how our highly adaptive neuro-musculo-skeletal system (NMSS) is (more or less progressively) supplanted by a less efficient and less adaptive system is an important challenge for aging research. In this respect, it is widely admitted that age-induced loss of behavioural adaptability emerges from the conjunction of various structural and functional modifications occurring in individual subsystems (cognitive, neural, sensorimotor, etc.). These modifications affect not only the individual functioning of each subsystem but also their interactions inducing reorganization in the whole NMSS [1-5]. The aim of the present study was to capture the consequences of these complex age-related changes at the level of behaviour by applying the framework of dynamical systems analysis to a functional sensorimotor task. This approach has been previously used to shed new light on how development modifies both the content and the use of the behavioural repertoire of movement patterns to adapt to task constraints. Accordingly, it has been shown that developmental change ensues from the reshaping of the infantile neuro-behavioural repertoire, wherein some existing patterns weaken or even finally disappear and new ones emerge [6]. In a lifespan perspective, whether or not this principle applies to aging/senescence has never been formally addressed elsewhere.

Inspired by dynamical systems theory, Kelso and colleagues (e.g., [7-11]) have proposed concepts and methods to account for how the coalition of various (neural, biomechanical, cognitive, etc.) constraints shapes the behavioural repertoire of movement patterns in coordination tasks. In this perspective, pattern dynamics (i.e. changes over time) are captured through the analysis of stability, loss of stability and transitions between coordination patterns (see [10,12] for details). For instance, co-existence of two stable coordination patterns, in-phase and anti-phase, in bimanual coordination (so called multi-stability), along with the transitions between them in response to increase in movement frequency, is highly functional since it allows short-term adaptations to task difficulty [8,9]. In older adults, it has been shown that bimanual coordination exhibits the same spontaneous dynamics as young adults except that phase transitions from the less stable (anti-phase) to the more stable (in-phase) coordination pattern occurs at a lower frequency [13,14]. Thus, in both young and older adults, multi-stability and flexibility of the dynamic repertoire of movement patterns can be considered as a mechanism permitting to preserve safety margin with respect to the constraints imposed on the NMSS. However, in older adults, pattern switching seemingly operates at lower levels of task constraints, thereby limiting the possibilities to further accommodate increasing demands. Such phenomenon can be a source of age-related loss of behavioural adaptability in most daily living activities.

The extension of these findings to unimanual tasks has been scarcely addressed in motor control literature. In this regard, it must be demonstrated that: i) for a given set of constraints, acting as a control parameter, the neuro-behavioural system spontaneously adopts a qualitatively distinguishable movement pattern and ii) when the value of this parameter is progressively increased the system switches from one movement organization to another. Providing empirical evidence supporting these phenomena requires the identification of: i) control parameter(s) triggering transitions between movement patterns and, ii) operating dynamic regimes that generate these patterns. The existence of different dynamic regimes, or generating mechanisms, as well as the occurrence of transitions in response to increasing task difficulty, has been reported in reciprocal unimanual tasks using the so-called Fitts’ paradigm [15,16]. In addition, we recently showed that the control of discrete rapid-aiming movements involves different movement patterns, presumably generated by different dynamic regimes, whereas the transition between them is triggered by the increase in task difficulty [17]. In the present work, we investigated if these patterns continue to exist in the neuro-behavioural repertoire of older adults, allowing functional adaptations over the short time scale of task-related constraints. Specifically, we explored age-related changes of movement patterns in a discrete target-directed rapid-aiming task, which involves functional movements requiring a speed-accuracy trade-off [18].

Fitts’ task is one of the most robust and reproducible paradigms to assess neuro-behavioural processes underlying speed-accuracy trade-off in different task situations (e.g., [19-25]) and populations (e.g., [26-29]). It consists in reaching as fast as possible to a target of a fixed width (W), placed at a given distance (D), either in a reciprocal (i.e. continuous movements between two targets; [30]) or a discrete manner (i.e. movements involving full stops on both home position and the target; [18]). In both cases, task difficulty is quantified by the index of difficulty (ID), which is calculated using the following formula [30]: ID=Log22×DW; where D is the distance between the home-position and the center of the target, and W is the target width. Accordingly, task difficulty can be scaled by increasing D and/or decreasing W. Since Fitts’ pioneering work [18,30], numerous studies have confirmed that movement time (MT) is linearly related to the ID according to the so-called Fitts’ law: *MT* = *α* + *β* × *ID*; where α and β are empirical constants depending on the individual and the task.

In both reciprocal and discrete Fitts’ tasks, it is currently admitted that the ID may act as a control parameter that triggers change in movement organization, in particular when it is manipulated via target width [15-17,31]. In this regard, it has been shown that when the ID was manipulated via accuracy constraints (W), rapid-aiming movements were controlled through two distinct dynamic regimes depending on the difficulty of the task [16,17]. The first regime was found to operate at lower levels of difficulty. At the level of kinematics, it was characterized by an increase of the duration of both acceleration (AT) and deceleration (DT) movement phases (a *co-variation pattern* between AT and DT) that is, the time elapsing before and after reaching peak velocity, respectively. The second regime was observed for higher IDs (>6 bits). It was characterized by a lengthening of DT, while AT remained unaffected by the difficulty increment (*dissociation pattern* between AT and DT). The discontinuity in the ID-AT relation observed around 6 bits was considered as a critical kinematic signature of the change in movement organization, which results from a transition between control mechanisms (as revealed by phase flow topology, see [16]). It was also shown to be concomitant with an increase in the variability of movement trajectory at target approach [16,17]. Overall, reported findings in Huys et al. [16] and Sleimen-Malkoun et al. [17] suggest that changes in dynamic regimes are associated with the presence of abrupt transitions in the kinematic organization of movements (see [17] for a discussion). According to the dynamical systems approach to coordination patterns, the switching toward a new (self-organizing) operating regime is presumably a means for (young) participants to preserve optimal accommodation to accuracy constraints of the task.

With regard to aging effects, most studies have shown that movement times of older participants increased more with the scaling of the ID than younger adults [4,32-35], especially in discrete aiming [36]. Specifically, elderly participants seem to spend more time in the neighbourhood of the target on the execution of corrective sub-movements in the deceleration movement phase (e.g., the movement portion following peak velocity). That finding has often been interpreted as an age-induced dependence on visual feedback during movement execution [37-39]. The significant effect of aging on the deceleration phase confers to it a central place in understanding age-related (loss of) adaptation. In this respect, a still unsettled debate concerns whether age-related kinematic changes result from functional limitations (e.g., general slowing hypothesis, see [4,40]), brain structure alterations (e.g., decrease in corticostriatal white matter connections, see [41]), or a deliberate strategic choice (e.g., favouring accuracy to the detriment of speed, see [42-45]). In the perspective of the dynamical systems approach, kinematic signatures observed in Fitts’ task are considered to reveal self-organizing neuro-behavioural patterns emerging as a result of the coalition of the multiple constraints arising from the task, the organism and the environment [16,17,46]. It remains however unknown whether pattern switching, which would indicate a transition between different dynamic regimes, is also observed in older adults. The objective of the present experiment was to explore this hypothesis by comparing behavioural dynamics of young and older adults in a discrete Fitts’ aiming task.

As a first step toward the exploration of dynamic adaptations to increasing task constraints in older adults, a necessary prerequisite was to refine the previously reported kinematic signatures of dynamic transitions in discrete aiming in young adults (see [17] for details). Therefore, we searched for a more specific indicator of AT/DT coordination. Specifically, we tested if the variability of AT/DT ratio can better characterize the switch between movement organizations. Our general hypothesis was that, in Fitts’ task, increasing the ID would trigger the transition toward a new AT/DT pattern. In addition, we predicted that: i) transition should occur at lower IDs in older as compared to younger adults (i.e. as previously observed in bimanual coordination, see [14]), and ii) it should be preceded by an increase in the variability of AT/DT ratio. According to the developmental literature (e.g., [6]), an alternative working hypothesis would be that the aging neuro-behavioural repertoire could lose one of its patterns. In that case, older adults would be expected to use the same pattern for all difficulty levels, specifically the one used by young participants at higher difficulty levels. If observed, it should be determined whether this process is adaptive (compensatory), that is, allowing older participants to maintain a good level of performance, at least for lower IDs. Correspondingly, one should observe a more pronounced aging effect for high levels of accuracy constraints (i.e. ID > 6 bits). Such observation would be in line with results observed in most neuro-cognitive aging studies (e.g., [47,48]). Indeed, the finding that, for an easy task, older adults use the pattern that young subjects adopt in more complicated tasks has already been reported in the literature. For example, the model of *Compensation-Related Utilization of Neural Circuits Hypothesis* (CRUNCH, [48]) was proposed to account for patterns of age-related over-activation and under-activation in cognitive tasks. The main idea is that elderly systematically use brain over-activation for less complex tasks, as a compensation process to maintain a performance that is comparable to that of younger adults.

## Methods

### Participants

Eighteen healthy volunteers were recruited for this study. They were subdivided into two groups: 9 young (mean age: 25 years; SD: 1.7; range: 23–28; 5 women) and 9 elderly adults (mean age: 80 years; SD: 5.4; range: 72–88; 5 women). All participants were self-reported as right-handed. Young participants self-declared that they did not suffer from neuro-muscular or uncorrected visual dysfunctions and that their right upper-limb was pain free. Elderly participants self-declared that they were healthy, physically active and autonomous in all of their daily living activities. Before the experimental session, they were assessed by a geriatrician to confirm that none of them suffered from cognitive or neuro-musculo-skeletal impairments that might bias the study.

All participants were unfamiliar with the task and the apparatus. They all provided written consent prior to the experiment. The protocol was approved by the ethic committee of Aix-Marseille University, and has therefore been in accordance with the ethical standards laid down in the Declaration of Helsinki.

### Experimental setup and procedure

A target-directed aiming experiment was carried out using a discrete Fitts’ task. During the experimental session, participants were comfortably seated on a chair at a height-adjusted table, with their left hand resting on their lap and their right forearm resting on a USB digitizer (Wacom Intuos4 XL; 1024 × 768 pixel resolution) positioned on the tabletop right in front of them, in portrait orientation. The digitizer was connected to a portable PC (Dell, Latitude-D420). Using a hand-held non-marking stylus (18 *g*; 156.5 × 14.9 *mm*; ~ 1 *mm* tip), participants were asked to perform discrete rapid-aiming movements, from the home position (a black *X* inside a .5 *mm* square) toward a target (red rectangle; 4 *cm* × W) placed underneath the transparent plastic film cover of the digitizer. Aiming movements involved shoulder flexion and elbow extension with full stops on home position (movement initiation) and the target (movement termination). Participants were firmly instructed to move as fast as possible without missing the target. To avoid trunk compensations, participants were required to keep their belly pressed against the table. The distance between home position and the centre of the target was kept fixed (*D* = 27 cm); nine targets with different width dimensions were used (6.8 ≤ *W* ≤ .3 *cm*), which yielded nine ID values (3.5 ≤ *ID* ≤ 7.5 *bits*). Details on experimental conditions can be found in Table 1. For each condition, participants were given three familiarization trials before performing the actually recorded trials. Task-conditions were randomly ordered and included fifteen self-paced repetitions each. When all trials were completed, the participants were given a short period to rest, during which, the experimenter replaced the target by a new one corresponding to the next ID condition to be performed. In all ID conditions, none of the participants missed the target for more than two trials. To make sure that young and older participants were not aiming to different areas of the target, hence not performing within the same scaling region of Fitts’ Law, we examined their effective behaviour by calculating the effective target width (We) on the basis of endpoints variance (see [22]). An effective index of difficulty (IDe) was calculated whenever the distribution of movement end points (centered on mean movement amplitude and bounded by the calculated We) was significantly different from the prescribed one (centered on target distance and bounded by target edges), otherwise the prescribed ID was kept. On average, the prescribed task was fairly respected in both groups, with a small exception for 3.5 bits condition in which an IDe had to be calculated for 3 young and 2 older participants. The mean IDe for this condition was 3.8 bits (SD = 0.7 for the young group, and 0.5 bits for elderly). Since a small number of participants (maximum of 3 subjects for the 3.5 condition) presented a significant difference between the prescribed and the effective target width, and that both groups were comparable for all difficulty levels, the initially prescribed IDs were kept.

**Table 1:** Experimental conditions

**Condition number**	**Width (cm)**	**Distance (cm)**	***ID *(bits)**
1	4.8	27.0	3.5
2	3.4	27.0	4.0
3	2.4	27.0	4.5
4	1.7	27.0	5.0
5	1.2	27.0	5.5
6	0.8	27.0	6.0
7	0.6	27.0	6.5
8	0.4	27.0	7.0
9	0.3	27.0	7.5

### Data acquisition and processing

Raw displacement time series of the pen-tip were acquired from the digitizer via custom made software at a sampling frequency of 250 Hz. The recorded data were processed using custom-written MATLAB (version 7.9.0, The MathWorks Inc.) routines. First, a second order low-pass Butterworth Filter with a cut-off frequency of 10 Hz and no phase shift was used to smooth time series. Then, velocity profiles were computed by numerically differentiating displacement data. Movement onset and offset were determined on the basis of the algorithm proposed by Teasdale et al. [49] using velocity profiles, with a critical velocity value of 0.04 × peak velocity. Acceleration and deceleration times (AT and DT) were defined as the duration prior to and following peak velocity, respectively. In case of more than one data point presenting the same maximal value, the first one was taken to calculate AT and DT.

### Dependent variables

We were interested in studying the effect of ID on: Movement time (MT); Acceleration time (AT); Deceleration time (DT); Variability of the relative timing between acceleration and deceleration phases (SD AT/DT) quantified by the standard deviation of AT/DT ratio in each condition for each participant; and Variability of movement trajectory in acceleration and deceleration movement phases as revealed by the velocity profile.

### Statistical analysis

#### Linear regressions

This part of the statistical analysis was conducted in MATLAB (The MathWorks Inc.). In accordance with Fitts’s law [18,30], the relation between ID and the temporal variables (MT, AT, DT) was analysed using the following linear equation: *Y* = *α* + *βX*; α and β terms and their respective Standard Errors were estimated using the simple Ordinary Least Square estimator. Mean Squared Error (MSE) and the coefficient of determination (R^2^) were also computed. The same method as in Sleimen-Malkoun et al. [17] was used to search for a discontinuity in ID-MT, ID-AT and ID-DT relations. First, the presence of an inflection in the non-parametric fitting curve of the corresponding data was visually inspected. The used smoothing spline algorithm is constructed with a smoothing parameter p, with a default value of .99, and it works by minimizing: p∑iyi−sxi2+1−p∫d2sdx22dx. Then, if an inflection was observed, a piecewise linear regression model with a combination of two linear relations was computed to test the presence of a breakpoint at the ID value around which the inflection was observed (see Figure 1 in [17] for an illustration). The piecewise model’s equation was the following: *Y*_*t*_ = (*α*_1_ + *β*_1_*X*_*t*_)*I*_1*t*_ + (*α*_2_ + *β*_2_*X*_*t*_)*I*_2*t*_; *I*_1*t*_ = 1 for *t* = 1;…; *j* otherwise *I*_1*t*_ = 0; and *I*_2*t*_ = 1 for *t* = *j* + 1;…; *k* otherwise *I*_2*t*_ = 0; *j* = number of observations up till the breakpoint, where *k* = total number of observations. R^2^ and MSE values of the piecewise model were compared to those of the simple linear regression model. The best model to fit the data was the one having the smallest MSE and the highest R^2^. A preliminary analysis of intra-individual behaviour showed comparable profiles between participants of each group. Regression analyses were hence applied to mean young and elderly data as it is the convention in aiming tasks.

**Figure 1:**
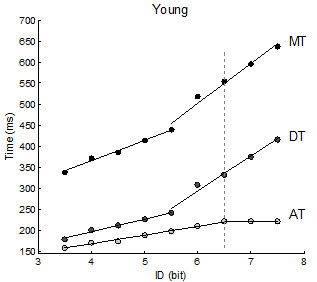
**Young participants’ mean temporal data with piecewise linear regression fittings.** The graph shows AT, DT and MT data of the young participants group fitted using the piecewise model. The dashed vertical line at 6.5 separates between the two movement organization patterns: before the line all three variables increased with ID, afterwards AT stopped being affected by ID.

Since the deceleration phase was considered to better reflect age-related changes, we paid particular interest to ID-DT relation for low (< 6 bits) and high ID levels. Two separate simple linear regressions were thus computed for each age-group, then, the slopes were compared using Student’s t statistics.

To relate between the performance of young and elderly, we used a Brinley plot [50], consisting in fitting the data points corresponding to young participants DTs in abscissa and elderly participants DTs in ordinates. This was done separately for low and high IDs (6–7.5 bits).

#### Principal component analysis

Principal component analysis (PCA) was used to study the variability of movement trajectory as revealed by the velocity profile (for a tutorial on the use of PCA to study variability, see [51]). This was done for movement portions prior to and after peak velocity that is, for acceleration and deceleration phases, respectively. This method allows the extraction of the smallest number of components (modes) that account for most of the variation in the original data without losing significant information. Since the eigenvalue of each mode reflects the amount of variance captured by it, the first mode’s eigenvalue was used to assess the degree of variability in the data (for more details on the application of this method to aiming tasks see [16,17]). Precisely, the closer the relative size of the eigenvalue of the first mode is to 1 the lesser is the inter-trial trajectory variability. This analysis was conducted in MATLAB for each participant in all conditions, then mean group values were calculated for each ID condition.

#### Analysis of variance

Analyses of variance were conducted in STATISTICA (StatSoft). All dependent variables were analysed with an Age (2) × ID (9) ANOVA with repeated measures on ID. When sphericity assumption was violated, Geisser-Greenhouse correction of degrees of freedom (*df*) was used. Accordingly, the reported *df* were rounded to the nearest whole number. The effect size was reported as: *η*^2^ = *SS*_*explained*_/*SS*_*total*_. The critical level for statistical significance was set to .05.

## Results

### Movement organization with increasing difficulty

Mean performance along with the statistics of ANOVA conducted on the temporal variables (MT, DT and AT) are summarized in Table 2. Results showed that, for all three variables, older adults had significantly longer movement durations (main effect of ID). MTs and DTs were lengthened by the ID increase independently of age (no significant Age × ID interactions). However, AT presented both significant ID main effect and Age × ID interaction (see Table 2). Decomposition of the interaction showed that ID only affected the young participants’ ATs.

**Table 2:** Performance characteristics and statistics of the temporal variables

	**Young**	**Elderly**	**ANOVA**			
***ID***	***M (SD)***	***M (SD)***	***Effect***	***df***	***F***	***η***^***2***^
*Movement Time (ms)*						
*3.5*	338 (73)	747 (272)				
*4.0*	372 (76)	754 (260)	Age***	1,16	18.25	.45
*4.5*	386 (90)	826 (248)	ID***	3,54	38.03	.11
*5.0*	415 (110)	840 (266)	*Age × ID*	4,56	.62	.001
*5.5*	439 (101)	895 (282)				
*6.0*	519 (133)	891 (286)				
*6.5*	555 (119)	964 (300)				
*7.0*	597 (106)	1008 (310)				
*7.5*	638 (123)	1082 (373)				
*Deceleration Time (ms)*						
*3.5*	179 (42)	367 (167)				
*4.0*	201 (44)	399 (188)	Age**	1,16	12.02	.32
*4.5*	212 (46)	431 (160)	ID***	3,41	45.62	.19
*5.0*	227 (64)	465 (192)	*Age × ID*	3,41	1.38	.006
*5.5*	242 (55)	520 (203)				
*6.0*	309 (82)	523 (208)				
*6.5*	333 (68)	586 (214)				
*7.0*	374 (76)	637 (241)				
*7.5*	416 (93)	692 (228)				
*Acceleration Time (ms)*						
*3.5*	159 (38)	380 (114)				
*4.0*	171 (40)	355 (78)	Age**	1,16	30.64	.60
*4.5*	175 (50)	395 (102)	ID*	4,62	3.06	.01
*5.0*	188 (51)	375 (78)	Age × ID*	4,62	2.84	.01
*5.5*	197 (54)	375 (87)				
*6.0*	210 (63)	368 (89)				
*6.5*	222 (56)	377 (100)				
*7.0*	222 (54)	371 (75)				
*7.5*	222 (52)	390 (114)				

M = mean; SD = Standard Deviation; *p < .05, **p < .01, ***p < .001.

**Figure 2:**
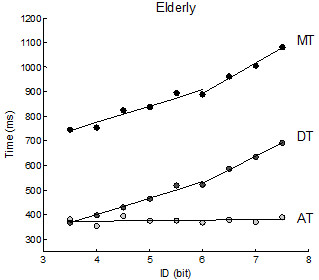
**Elderly participants’ mean temporal data with piecewise linear regression fittings.** The graph shows AT, DT and MT data of the older participants group fitted using the piecewise model. Note that the piecewise fitting did not account for ID-AT relation, thus the simple linear fitting is shown although it was also not significant (R^2^ = .03). Both MT and DT increased with ID.

In both age groups, regression analyses were used to determine whether different patterns of movement organization were used across ID conditions. The estimates of simple linear regressions are reported in Table 3. In young participants, Fitts’ law accounted for the effect of ID on MT, AT and DT. However, in the elderly group, it only accounted for changes in MT and DT (ID-AT relation was not significant; see Table 3). Further analyses using the piecewise linear regression model revealed discontinuities in the relations between the ID and the temporal variables. In the group of young participants a breakpoint was identified at 5.5 bits for MT and DT, and at 6.5 bits for AT (Table 4; Figure 1). In the elderly, a breakpoint was detected for only MT and DT at 6 bits (Table 4, Figure 2).

**Table 3:** Simple linear regressions’ estimates for mean data of young and elderly participants

	**Young**			**Elderly**		
	**MT**	**AT**	**DT**	**MT**	**AT**	**DT**
*R*^*2*^	.97	.95	.95	.96	.03	.98
*MSE*	247.90	213.60	280.72	497.36	151.78	238.08
*α*	49.05	100.44	−51.30^§^	444.21^§^	368.35	75.86^§^
*β*	77.12	17.42	59.70	80.97	1.41^§^	79.55

^§^p > .05.

**Table 4:** Piecewise linear regressions’ estimates for mean data of young and elderly participants

	**Young**			**Elderly**	
	**MT**	**AT**	**DT**	**MT**	**DT**
*R*^*2*^	.99	.99	.98	.97	.98
*MSE*	102.68	28.08	204.78	306.25	108.16
*α*_*1*_*/α*_*2*_	170.19/-67.12^§^	110.4/225.10	76.47/-202.86^§^	467.9/154.9^§^	100.7/-143
*β*_*1*_*/ β*_*2*_	48.86/94.87	15.73/-0.44^§^	30.14/82.72	76.5/123.14	74.57/111.52

^§^p > .05.

**Figure 3:**
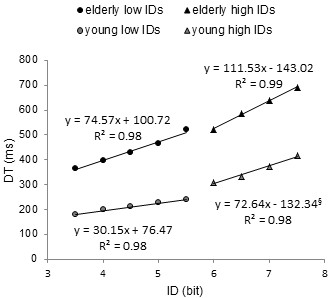
**Decomposition of ID-DT relation as function of ID range and age group.** Circles represents low IDs and triangles high IDs. Grey markers are used for young participants black markers for elderly. Mean data, linear fittings and their corresponding equations are presented. The “§” marked coefficient was not significant at 5%. It can be seen how DT is affected differently by ID at low and high difficulty levels and between age groups. Interestingly, the slope value for low IDs in older participants is found to be comparable to that for high IDs in younger participants.

**Figure 4:**
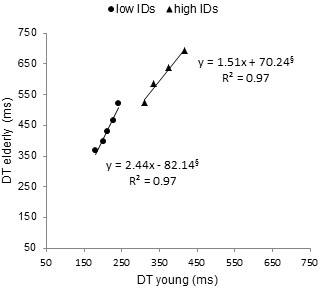
**Brinley plots for DTs observed at lower and higher ID ranges.** Mean DT data, linear fittings and their corresponding equations are presented for low (circles) and high (triangle) ID ranges. The “§”marked coefficients were not significant at 5%. The slope of the Brinley function quantifying age related slowing is greater for lower than for higher IDs.

The analysis of DTs in young and older participants for low (3.5-5.5 bits) and high (6–7.5 bits) ID levels revealed that slopes of regression functions were significantly different between groups for the same ID range (p < .001). However, similar slope values, with no statistically significant difference (p > .05), were observed in older participants at low IDs and in young participants at high IDs (74.57 and 72.64 respectively, Figure 3). Brinley plot analysis (Figure 4) showed that slowing rates as measured by the slopes of the linear regression function were significantly higher for low ID values than for higher IDs (2.4 and 1.5 for lower and higher ID ranges, respectively). In addition, both slopes were significantly different from 1 (p < .005).

### Variability of movement trajectory

**Figure 5:**
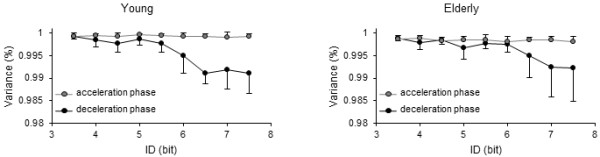
**PCA results.** Variance captured by the first mode for acceleration (grey markers) and deceleration (black markers) movement phases in both age groups. Error bars represent one standard deviation from the mean. The same pattern can be observed for both groups: movement trajectory presented a low and stable variability throughout the difficulty range in the acceleration phase, however, fluctuations progressively increased in the deceleration phase (starting 5.5 bits in young and 6 bits in elderly participants).

PCA performed on velocity profiles for acceleration and deceleration movement phases showed comparable patterns between the two groups (Figure 5). For the deceleration phase, the percentage of variance explained by the first eigenvalue progressively decreased after 5.5 bits for the young group and after 6 bits for the elderly, which indicated that movement trajectories became more variable after these ID values. On the other hand, for both groups, the variance of movement trajectory in the acceleration phase remained rather stable across ID conditions.

Analyses of variance were conducted separately on acceleration and deceleration phases. For the deceleration phase, the ANOVA (Age × ID) showed a main effect of ID (*F*(3,46) = 17.4, *p* < .001, *η*^2^ = .4), but no significant effects were found for age (*F*(1,16) = .53, *p* > .05, *η*^2^ = .006) and Age × ID interaction (*F*(3,46) = 1.43, *p* > .05, *η*^2^ = .03). For the acceleration phase, only a significant effect of Age (*F*(1,16) = 17.27, *p* < .001, *η*^2^ = .25) was found, with the elderly being more variable. No significance was found for ID main effect (*F*(4,72) = 1.23, *p* > .05, *η*^2^ = .03) and Age × ID interaction (*F*(4,72).76, *p* > .05, *η*^2^ = .02).

### Variability of AT/DT ratio

**Figure 6:**
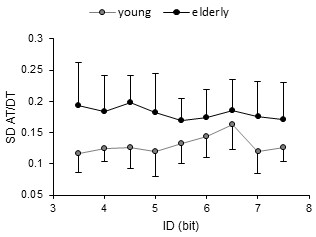
**Variability of AT/DT ratio across ID conditions.** Grey markers represent young participants’ data and black ones elderly’s data. Error bars represent one standard deviation from the mean. Only the ratio of young participants shows a distinguishable pattern of change across ID levels, with a peak at 6.5 bits where the discontinuity was detected in ID-AT relation followed by a decrease afterwards.

The variability of AT/DT ratio across ID levels presented different patterns in the two age groups (Figure 6). In older participants, AT/DT ratio remained unchanged. Conversely, in young participants, it presented a progressive increase starting 5 bits up-to 6.5 bits, and it abruptly decreased afterwards. A one factor repeated measures ANOVA was done to verify if ID scaling had a significant effect in each participants group. This analysis confirmed the absence of effect in older participants (*F*(8,64) = .35, *p* > .05, *η*^2^ = .03), while it showed a significant effect in young participants (*F*(8,64) = 2.12, *p* < .05, *η*^2^ = .17).

## Discussion

In the present study, we explored age-related changes in movement patterns in response to increasing task constraints in discrete Fitts’ task. We predicted that, compared to young adults, in older adults, task-induced transitions between behavioural patterns would either occur for a much lower difficulty level or remain not observed for the whole ID range. In other terms, elderly would systematically be using the movement pattern that young participants only use for more complicated tasks (i.e. higher ID). According to some recent work on rapid aiming [16,17], our working hypothesis was that the presence of different dynamic regimes and movement patterns in Fitts’ task would be related to how acceleration and deceleration times are affected by the increase of task difficulty. Accordingly, we expected the transitions between these patterns to be also accompanied by concomitant changes in variability of AT/DT ratio. In this context, we were interested in determining: i) whether behavioural flexibility, that is the capability of switching between different movement organizations, is preserved in older adults, and ii) whether such flexibility continues to be adaptive, that is contributing in maintaining task performance levels, at least at low difficulty values.

Results observed in young participants replicated those previously observed by Sleimen-Malkoun et al. [17]. They showed that, in a discrete Fitts’ task, young adults used different movement patterns to accommodate increasing ID levels before and after a transition threshold located around 6.5 bits. The transition was marked by: i) the presence of a discontinuity in ID-AT relation (at 6.5 bits, Figure 1) revealing the switch from a *co-variation* to a *dissociation* pattern between ID-AT and ID-DT, and ii) an increase in the variability of movement trajectory in the deceleration phase (starting at 5.5 bits, Figure 5, left panel). The analysis of variability of AT/DT ratio, which was not previously used in Sleimen-Malkoun et al.’s study [17], improved the interpretation drawn from our previous and current findings. Indeed, this variable was sensitive to the destabilization of the adopted AT/DT pattern. Specifically, the variability of AT/DT ratio showed a progressive increase between 5 and 6.5 bits, followed by an abrupt decrease (Figure 6), which perfectly paralleled ID-AT discontinuity. Such pattern of changes in variability can be considered as a strong signature of the occurrence of a transition that is, in present task situation, a switch from the *dissociation* toward the *co-variation* movement pattern. On the other hand, the increase in the variability of movement trajectory of the deceleration phase (as revealed by PCA) was accompanied with changes in the slope of ID-MT and ID-DT relations (after 5.5 bits). These changes were not found to be significant in mean group data analysis in Sleimen-Malkoun et al.’s study [17]. Although, this finding is not completely new, it has not however been extensively discussed in the literature. Indeed, the currently observed changes of slope are consistent with Gan and Hoffmann’s [52] predictions and results. Their findings were interpreted as evidence that, depending on ID level and on the prescribed amplitude of the movement (i.e. D value), movement control would involve more or less ballistic or feedback driven processes, and thus Fitts’ law would more or less apply to certain difficulty ranges [52,53].

The present results afford a quite different and more precise picture of task-related adaptations than those reported in previous studies [16,17]. In particular, the ID-AT discontinuity, revealing the change in the adopted movement organization, seemed to be closely related to the abrupt change in the variability of AT/DT ratio (after 6.5 bits). Conversely, the change in slope of ID-MT and ID-DT relations was shown to be concomitant to the progressive increase in the variability of movement trajectory (starting at 5.5 bits). These results suggest that changes observed in ID-AT are relatively independent from those observed in ID-MT and ID-DT relations, which was also supported by the comparison between young and older participants.

Indeed, in older adults, although MT, AT and DT were always longer as compared to young participants, ID-MT and ID-DT relations remained comparable between the two age groups. Conversely, ID-AT relation presented a different pattern between the two groups of participants: in young participants, ID affected ATs up-till 6.5 bits, while in elderly no significant ID-AT relation was found throughout the entire range of difficulty. Interestingly, scaling the ID did not affect the variability of AT/DT ratio in older adults, while it affected the variability of movement trajectory at target approach similarly in both groups (with a slight shift of .5 bits; see PCA results and Figure 5). These findings suggest that some kinematic adaptations to increasing task difficulty are not altered during aging, while others disappear. As a consequence, in elderly, only a single pattern of movement organization would be used over the whole range of ID levels that is, independently of task difficulty. Strikingly, the movement pattern used by older participants was the *dissociation* pattern, which younger participants used only at high levels of task difficulty. This suggests that, at least in older adults, the functional use of a movement pattern is not closely linked to the objective difficulty level of the task. Instead, it might be hypothesized that, in older adults, there are less available resources within the neuro-behavioural system of older adults to assemble, or switch toward, the *dissociation* pattern, thereby leading to functional limitations that should inevitably manifest in task performance. The mechanism at origin of such *compression* of the neurobehavioural repertoire remains unknown. A plausible hypothesis is that the *co-variation* pattern disappeared (or became simply inaccessible) from the task-specific repertoire of elderly as a result of the conjunction of muscular, cognitive and sensorimotor changes occurring in the aging NMSS. Subsequently, the initially task-specific multi-stable neurobehavioural repertoire became mono-stable.

Analyses of the slopes of efficiency and Brinley functions conducted on DTs provided strong support to the hypothesis of an age-related repertoire compression. Indeed, ID-DT relation presented a similar slope in older adults for low IDs (74.57) and in young participants for high IDs (72.64) (see Figure 3), likely as a consequence of using the same movement pattern. In addition, for high ID levels, the ID-DT slope observed in older participants was significantly greater than those observed at low ID levels (111.53 *ms* and 75.57 *ms*, respectively) and greater that those observed in young participants for the same ID range (72.64 *ms*). When translated into relative slowing between young and older adults through Brinley plots analysis, such differences in ID-MT slopes led to a more pronounced multiplicative slowing for low ID levels (2.44 for IDs < 6 *bits*) than for higher levels (1.51; see Figure 4). This observation is somewhat counterintuitive. Indeed, according to the classical Age × Complexity effect [54], one would expect that for higher IDs the aging neuro-behavioural system will be even more overloaded by task constraints, thereby leading to a greater slowing ratio. Our analyses revealed however that the deficit of older participants in terms of information processing and movement control was more pronounced for the easier ID conditions. In addition, it is noticeable that the slowing ratio observed at low ID levels was significantly larger than those generally observed in previous studies examining the entire ID range [4,55]. All these findings suggest that, in older adults, the use of the *dissociation* pattern is inadequate and detrimental to task performance (i.e. yielding longer MTs) at low ID levels. It also adds questions to the current debate about the compensatory versus non-compensatory nature of the observed neuro-behavioural adaptations. For instance, in recent models of neuro-plasticity of the aging brain, neural over-activation patterns in cognitive tasks performed by elderly are considered of compensatory nature (see [55] for an overview). In particular, it is generally admitted that, for tasks of low difficulty, older adults preserve close levels of performance to young adults by using greater levels of brain activation [48,56]. Nevertheless, at high levels of difficulty, loss of adaptation and dramatic decrease in (cognitive) performance are observed, presumably because further activation, and thus compensation, becomes impossible. The present results afford a quite different picture. Indeed, they suggest that the adoption of the dissociation pattern by older participants was not compensatory at lower difficulty values. In the lack of concomitant brain imaging measures in the present study, it is impossible to establish a clear relationship between brain and behaviour. Nevertheless, our results suggest that compensation is not the rule in age-related changes in neuro-behavioural patterns, at least in sensorimotor tasks.

Since Fitts’ law has repeatedly shown its weakness in accounting for MT when ID decreases below 3 bits (e.g., [52,56]) very low ID values were purposely excluded from the experimental setting. Nevertheless, one could wonder what would happen if the task was extremely easy, that is whether older subjects could exhibit the initial *co-variation* pattern and whether they would present a lower slowing ratio. Theoretically speaking, two scenarios can be envisaged: i) if the co-variation pattern is still available in the elderly’s repertoire, one should expect to observe the co-variation pattern for very easy tasks before prematurely switching toward the dissociation pattern; ii) if the co-variation pattern disappeared from the elderly’s repertoire, one should expect to continue to observe the dissociation pattern independently of the difficulty level.

The question also remains on the functional meaning and the mechanisms behind the lack of accessibility to multiple movement patterns in old age. On the one hand; it could be hypothesized that some patterns existing in the neuro-behavioural repertoire are lost, independently of the task to be performed. On the other hand, the impoverishment of the repertoire could be attributed to a change in the coalition of constraints arising from multiple origins (i.e. organism- and task-related constraints) that finally precludes the emergence of multiple patterns. These are two different views of pattern formation. In the former, constraints related to the NMSS are considered to be dominant (or even genetically programmed), thereby leading to more or less progressive loss of the pre-existing movement patterns trapped in the NMSS as the result of the aging process. In the latter perspective, movement patterns are considered to emerge in real-time from a coalition of constraints that is, from the interaction between Fitts’ task and intrinsic constraints/principles to the NMSS (as are muscle homology principle or symmetry principles in bimanual coordination). According to this view, assembling of movement patterns is a multi-causal phenomenon, in which target width presumably plays a critical role in conjunction with evolving cognitive and motor capabilities of the (aging) NMSS. Thus, the change in the availability of multiple movement patterns in discrete Fitts’ task is consistent with the idea of emergence as an on-going process in the whole brain-body-environment system. Nevertheless, age-related adaptations observed in discrete Fitts’ task were difficult to compare to those previously observed in bimanual coordination studies (e.g., [14]) due to differences between unimanual aiming and bimanual coordination tasks. Specifically, in bimanual coordination tasks, young adults exploited intrinsic (in-phase and anti-phase) patterns, reflecting spontaneous tendencies of the NMSS to activate simultaneously homologous and non-homologous muscles groups quite independently of the nature of the task [8,9]. These intrinsic patterns might be resistive to age-related biomechanical, muscular, or informational changes, and thus persist in elderly. In discrete Fitts’ task however, no such intrinsic patterns exist. Instead, the used movement patterns are temporarily assembled as the result of available muscular, cognitive and sensorimotor resources under task-dependent constraints (i.e. physical dimensions that are manipulated to increase the ID). Consequently, these movement patterns might be more sensitive to age-related changes in neuro-muscular, cognitive and sensorimotor processes.

## Conclusions

In the present work, we adopted a dynamical systems-inspired perspective to investigate age-related changes of movement patterns in discrete Fitts’ task. On the one hand, results observed in young participants contributed to improving the identification of the typical kinematic signatures that are directly related to the transition in movement patterns as task difficulty increases. On the other hand, results observed in older adults suggested the presence of *non-adaptive* age-related *compression* of the behavioural repertoire. At least, the present findings do lend credence to the heuristic value of the dynamical systems approach to explore how the repertoire of movement patterns evolves with aging and how it is exploited to adapt to increase in task difficulty. Nonetheless, the dynamics of movement patterns in discrete Fitts’ task is far from being completely elucidated. In particular, quantitative modelling of the dynamic regimes responsible of the generation of discrete target-directed aiming movements under different accuracy constraints remains to be done. Indeed, a limit of the present work is that no conventional dynamic analyses were used, relating movement reorganization to a change in the underlying control mechanisms.

Overall, the present results add to the interest and to the challenge of establishing solid knowledge about the dynamic processes and mechanisms underlying both movement production and control in advanced age.

## Competing interests

The authors declare that they have no competing interests.

## Authors’ contributions

RSM conceived, designed and carried out the experiment. She also analysed the data and was the lead writer of this manuscript. JJT contributed to the study design, data interpretation and writing of the manuscript. EB reviewed the first draft of the manuscript. All authors have read and approved the final version of the manuscript.
